# 2-Methyl-3-(*n*-octylsulfan­yl)quinoxaline

**DOI:** 10.1107/S1600536810011542

**Published:** 2010-03-31

**Authors:** Youssef Ramli, Rachid Slimani, Hafid Zouihri, Saïd Lazar, E. M. Essassi

**Affiliations:** a Laboratoire Nationale de Contrôle des Médicaments, Direction du Médicament et de la Pharmacie, BP 6206, 10000 Rabat, Morocco; b Laboratoire de Biochimie, Environnement et Agroalimentaire (URAC 36), Faculté des Sciences et Techniques Mohammedia, Université Hassan II Mohammedia-Casablana, BP 146, 20800 Mohammedia, Morocco; c Laboratoires de Diffraction des Rayons X, Division UATRS, Centre National pour la Recherche Scientifique et Technique, Rabat, Morocco; d Laboratoire de Chimie Organique Hétérocyclique, Université Mohammed, V-Agdal, BP 1014, Rabat, Morocco

## Abstract

All the non-H atoms of the title compound, C_17_H_24_N_2_S, lie almost in a common plane (r.m.s. deviation = 0.049 Å). The octyl chain adopts an all-*trans* conformation.

## Related literature

For the biological activity of quinoxaline derivatives, see: Kleim *et al.* (1995 ▶). For the anti­tumor and anti­tuberculous properties of quinoxaline derivatives, see: Abasolo *et al.* (1987 ▶); Rodrigo *et al.* (2002 ▶). For the anti­fungal, herbicidal, anti­dyslipidemic and anti-oxidative activity of quinoxaline derivatives, see: Jampilek *et al.* (2005 ▶); Sashidhara *et al.* (2009 ▶); Watkins *et al.* (2009 ▶).
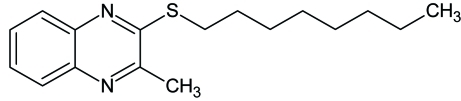


## Experimental

### 

#### Crystal data

C_17_H_24_N_2_S
*M* *_r_* = 288.44
Triclinic, 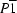

*a* = 7.3514 (3) Å
*b* = 8.2978 (3) Å
*c* = 14.2168 (5) Å
α = 92.275 (2)°
β = 98.706 (2)°
γ = 103.810 (2)°
*V* = 829.86 (5) Å^3^
*Z* = 2
Mo *K*α radiation
μ = 0.19 mm^−1^
*T* = 296 K
0.26 × 0.17 × 0.16 mm

#### Data collection

Bruker APEXII CCD detector diffractometer
29319 measured reflections
6513 independent reflections
3251 reflections with *I* > 2σ(*I*)
*R*_int_ = 0.046

#### Refinement

*R*[*F* ^2^ > 2σ(*F* ^2^)] = 0.054
*wR*(*F* ^2^) = 0.161
*S* = 1.00
6513 reflections
183 parameters
H-atom parameters constrained
Δρ_max_ = 0.24 e Å^−3^
Δρ_min_ = −0.21 e Å^−3^

### 

Data collection: *APEX2* (Bruker, 2005 ▶); cell refinement: *SAINT* (Bruker, 2005 ▶); data reduction: *SAINT*; program(s) used to solve structure: *SHELXS97* (Sheldrick, 2008 ▶); program(s) used to refine structure: *SHELXL97* (Sheldrick, 2008 ▶); molecular graphics: *XP* (Sheldrick, 2008 ▶); software used to prepare material for publication: *publCIF* (Westrip, 2010 ▶).

## Supplementary Material

Crystal structure: contains datablocks I, global. DOI: 10.1107/S1600536810011542/bt5230sup1.cif

Structure factors: contains datablocks I. DOI: 10.1107/S1600536810011542/bt5230Isup2.hkl

Additional supplementary materials:  crystallographic information; 3D view; checkCIF report

## Figures and Tables

**Fig. 1.**
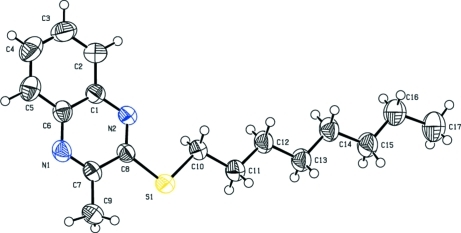
: Molecular structure of the title compound with the atom-labelling scheme. Displacement ellipsoids are drawn at the 50% probability level. H atoms are represented as small spheres of arbitrary radii.

## supplementary crystallographic information

### Comment

Quinoxaline derivatives are used as starting compounds in the synthesis of various more complex heterocyclic systems. On the other hand, quinoxaline core constitutes a structural fragment of many important pharmaceuticals and biologically active substances so that compounds containing a quinoxaline fragment attract strong interest of synthetic chemists and biochemists. Quinoxaline derivatives were found to exhibit antimicrobial (Kleim *et al.* 1995 ), antitumor (Abasolo *et al.*, 1987), and antituberculous activity (Rodrigo *et al.*, 2002).

Bond lengths and angles in title molecule (Fig.1) are normal.

### Experimental

To a solution of 3-methylequinoxaline-2(1*H*)-thione (1 g, 5.68053 mmol) in dimethylformamide (20 ml), was added CH_3_(CH_2_)_6_C_2_I ,K_2_CO_3_ (1 g, 7.46 mmol) and a catalytic quantity of tetrabutylammoniumbromide. The mixture was stirred at room temperature for 24 h. The solution was filtered to remove the salts. The solvent was removed under reduced pressure.

The residue was crystallized in ethanol to afford the title compound as colourless crystals.

### Refinement

All H atoms were geometrically positioned and treated as riding with C_methyl_—H = 0.96 Å, C_methylene_—H = 0.97 Å and C_aromatic_—H = 0.93 Å with *U*(H) = 1.2*U*_eq_(C) or *U*(H) = 1.5*U*_eq_(C_methyl_) .

### Crystal data

**Table:**

C_17_H_24_N_2_S	*Z* = 2
*M**_r_* = 288.44	*F*(000) = 312
Triclinic, *P*1	*D*_x_ = 1.154 Mg m^−^^3^
Hall symbol: -P 1	Melting point: 374 K
*a* = 7.3514 (3) Å	Mo *K*α radiation, λ = 0.71073 Å
*b* = 8.2978 (3) Å	Cell parameters from 2685 reflections
*c* = 14.2168 (5) Å	θ = 2.5–27.3°
α = 92.275 (2)°	µ = 0.19 mm^−^^1^
β = 98.706 (2)°	*T* = 296 K
γ = 103.810 (2)°	Block, colourless
*V* = 829.86 (5) Å^3^	0.26 × 0.17 × 0.16 mm

### Data collection

**Table:**

Bruker APEXII CCD detector diffractometer	3251 reflections with *I* > 2σ(*I*)
Radiation source: fine-focus sealed tube	*R*_int_ = 0.046
graphite	θ_max_ = 33.6°, θ_min_ = 2.8°
ω and φ scans	*h* = −10→11
29319 measured reflections	*k* = −12→12
6513 independent reflections	*l* = −22→22

### Refinement

**Table:**

Refinement on *F*^2^	Primary atom site location: structure-invariant direct methods
Least-squares matrix: full	Secondary atom site location: difference Fourier map
*R*[*F*^2^ > 2σ(*F*^2^)] = 0.054	Hydrogen site location: inferred from neighbouring sites
*wR*(*F*^2^) = 0.161	H-atom parameters constrained
*S* = 1.00	*w* = 1/[σ^2^(*F*_o_^2^) + (0.0769*P*)^2^ + 0.0189*P*] where *P* = (*F*_o_^2^ + 2*F*_c_^2^)/3
6513 reflections	(Δ/σ)_max_ < 0.001
183 parameters	Δρ_max_ = 0.24 e Å^−^^3^
0 restraints	Δρ_min_ = −0.21 e Å^−^^3^

### Special details

**Table:**

Experimental. The data collection nominally covered a sphere of reciprocal space, by a combination of seven sets of exposures; each set had a different φ angle for the crystal and each exposure covered 0.5° in ω and 25 seconds in time. The crystal-to-detector distance was 37.5 mm.
Geometry. All esds (except the esd in the dihedral angle between two l.s. planes) are estimated using the full covariance matrix. The cell esds are taken into account individually in the estimation of esds in distances, angles and torsion angles; correlations between esds in cell parameters are only used when they are defined by crystal symmetry. An approximate (isotropic) treatment of cell esds is used for estimating esds involving l.s. planes.
Refinement. Refinement of *F*^2^ against ALL reflections. The weighted *R*-factor wR and goodness of fit *S* are based on *F*^2^, conventional *R*-factors *R* are based on *F*, with *F* set to zero for negative *F*^2^. The threshold expression of *F*^2^ > σ(*F*^2^) is used only for calculating *R*-factors(gt) etc. and is not relevant to the choice of reflections for refinement. *R*-factors based on *F*^2^ are statistically about twice as large as those based on *F*, and *R*- factors based on ALL data will be even larger.

### Fractional atomic coordinates and isotropic or equivalent isotropic displacement parameters (Å^2^)

**Table:**

	*x*	*y*	*z*	*U*_iso_*/*U*_eq_	
S1	0.61857 (5)	0.28251 (5)	0.04284 (3)	0.05291 (14)	
N2	0.67349 (16)	0.13786 (14)	−0.11823 (8)	0.0467 (3)	
C8	0.54850 (18)	0.17628 (16)	−0.07085 (10)	0.0426 (3)	
N1	0.28116 (16)	0.05407 (15)	−0.19181 (9)	0.0518 (3)	
C10	0.87038 (19)	0.29990 (18)	0.06244 (11)	0.0493 (3)	
H10A	0.8933	0.1896	0.0597	0.059*	
H10B	0.9263	0.3596	0.0124	0.059*	
C7	0.34717 (18)	0.13428 (17)	−0.10860 (11)	0.0472 (3)	
C1	0.6056 (2)	0.05508 (17)	−0.20663 (10)	0.0476 (3)	
C11	0.9631 (2)	0.39073 (19)	0.15840 (11)	0.0552 (4)	
H11A	0.9086	0.3298	0.2085	0.066*	
H11B	0.9379	0.5002	0.1615	0.066*	
C6	0.4093 (2)	0.01226 (17)	−0.24318 (10)	0.0503 (3)	
C12	1.1760 (2)	0.40888 (19)	0.17481 (11)	0.0550 (4)	
H12A	1.1990	0.2989	0.1689	0.066*	
H12B	1.2287	0.4711	0.1247	0.066*	
C14	1.4944 (2)	0.5180 (2)	0.27832 (11)	0.0593 (4)	
H14A	1.5201	0.4097	0.2689	0.071*	
H14B	1.5369	0.5831	0.2270	0.071*	
C13	1.2816 (2)	0.4944 (2)	0.27024 (11)	0.0571 (4)	
H13A	1.2372	0.4285	0.3209	0.069*	
H13B	1.2539	0.6022	0.2786	0.069*	
C5	0.3444 (3)	−0.0724 (2)	−0.33399 (12)	0.0654 (4)	
H5	0.2151	−0.1026	−0.3580	0.078*	
C15	1.6108 (2)	0.6024 (2)	0.37177 (12)	0.0659 (4)	
H15A	1.5757	0.5341	0.4231	0.079*	
H15B	1.5811	0.7085	0.3835	0.079*	
C9	0.2124 (2)	0.1842 (2)	−0.05151 (13)	0.0626 (4)	
H9A	0.0860	0.1513	−0.0872	0.094*	
H9B	0.2480	0.3028	−0.0377	0.094*	
H9C	0.2166	0.1308	0.0072	0.094*	
C2	0.7318 (3)	0.0135 (2)	−0.26280 (12)	0.0630 (4)	
H2	0.8616	0.0409	−0.2397	0.076*	
C16	1.8230 (3)	0.6316 (3)	0.37289 (14)	0.0818 (6)	
H16A	1.8523	0.5247	0.3632	0.098*	
H16B	1.8562	0.6956	0.3196	0.098*	
C3	0.6642 (3)	−0.0667 (2)	−0.35107 (13)	0.0737 (5)	
H3	0.7489	−0.0927	−0.3880	0.088*	
C4	0.4702 (3)	−0.1106 (2)	−0.38715 (13)	0.0748 (5)	
H4	0.4266	−0.1660	−0.4475	0.090*	
C17	1.9438 (3)	0.7205 (3)	0.46263 (17)	0.1066 (8)	
H17A	1.9224	0.8295	0.4708	0.160*	
H17B	2.0752	0.7303	0.4587	0.160*	
H17C	1.9114	0.6589	0.5160	0.160*	

### Atomic displacement parameters (Å^2^)

**Table:**

	*U*^11^	*U*^22^	*U*^33^	*U*^12^	*U*^13^	*U*^23^
S1	0.0394 (2)	0.0656 (2)	0.0517 (2)	0.01121 (16)	0.00697 (15)	−0.00649 (16)
N2	0.0370 (6)	0.0531 (6)	0.0480 (7)	0.0077 (5)	0.0075 (5)	0.0010 (5)
C8	0.0345 (7)	0.0467 (7)	0.0450 (7)	0.0078 (5)	0.0052 (5)	0.0039 (6)
N1	0.0393 (6)	0.0597 (7)	0.0529 (7)	0.0106 (5)	−0.0004 (5)	0.0030 (6)
C10	0.0370 (7)	0.0551 (8)	0.0511 (8)	0.0046 (6)	0.0052 (6)	−0.0026 (6)
C7	0.0346 (7)	0.0498 (7)	0.0555 (9)	0.0087 (6)	0.0049 (6)	0.0064 (6)
C1	0.0454 (8)	0.0501 (7)	0.0467 (8)	0.0093 (6)	0.0090 (6)	0.0041 (6)
C11	0.0465 (8)	0.0620 (9)	0.0523 (9)	0.0077 (7)	0.0041 (7)	−0.0011 (7)
C6	0.0489 (8)	0.0518 (8)	0.0468 (8)	0.0095 (6)	0.0018 (6)	0.0059 (6)
C12	0.0475 (8)	0.0601 (8)	0.0507 (8)	0.0058 (7)	0.0004 (7)	0.0006 (7)
C14	0.0528 (9)	0.0644 (9)	0.0518 (9)	0.0041 (7)	−0.0018 (7)	0.0044 (7)
C13	0.0518 (9)	0.0618 (9)	0.0514 (9)	0.0071 (7)	0.0009 (7)	0.0010 (7)
C5	0.0667 (11)	0.0707 (10)	0.0515 (9)	0.0130 (8)	−0.0048 (8)	−0.0018 (8)
C15	0.0579 (10)	0.0745 (10)	0.0554 (9)	0.0070 (8)	−0.0045 (8)	−0.0010 (8)
C9	0.0383 (8)	0.0779 (10)	0.0721 (11)	0.0181 (7)	0.0082 (7)	−0.0061 (8)
C2	0.0597 (10)	0.0699 (10)	0.0616 (10)	0.0151 (8)	0.0203 (8)	−0.0006 (8)
C16	0.0637 (12)	0.1066 (15)	0.0644 (11)	0.0139 (10)	−0.0064 (9)	−0.0109 (10)
C3	0.0848 (14)	0.0779 (11)	0.0645 (11)	0.0220 (10)	0.0298 (10)	−0.0013 (9)
C4	0.0960 (15)	0.0749 (11)	0.0481 (10)	0.0159 (10)	0.0067 (10)	−0.0051 (8)
C17	0.0735 (14)	0.138 (2)	0.0857 (16)	0.0068 (13)	−0.0159 (12)	−0.0248 (14)

### Geometric parameters (Å, °)

**Table:**

S1—C8	1.7530 (14)	C14—H14B	0.9700
S1—C10	1.7995 (14)	C13—H13A	0.9700
N2—C8	1.3077 (18)	C13—H13B	0.9700
N2—C1	1.3705 (18)	C5—C4	1.363 (3)
C8—C7	1.4485 (18)	C5—H5	0.9300
N1—C7	1.2990 (18)	C15—C16	1.518 (2)
N1—C6	1.373 (2)	C15—H15A	0.9700
C10—C11	1.5119 (19)	C15—H15B	0.9700
C10—H10A	0.9700	C9—H9A	0.9600
C10—H10B	0.9700	C9—H9B	0.9600
C7—C9	1.491 (2)	C9—H9C	0.9600
C1—C2	1.405 (2)	C2—C3	1.361 (2)
C1—C6	1.411 (2)	C2—H2	0.9300
C11—C12	1.517 (2)	C16—C17	1.493 (2)
C11—H11A	0.9700	C16—H16A	0.9700
C11—H11B	0.9700	C16—H16B	0.9700
C6—C5	1.403 (2)	C3—C4	1.395 (3)
C12—C13	1.513 (2)	C3—H3	0.9300
C12—H12A	0.9700	C4—H4	0.9300
C12—H12B	0.9700	C17—H17A	0.9600
C14—C15	1.511 (2)	C17—H17B	0.9600
C14—C13	1.515 (2)	C17—H17C	0.9600
C14—H14A	0.9700		
			
C8—S1—C10	101.52 (7)	C14—C13—H13A	109.2
C8—N2—C1	116.84 (12)	C12—C13—H13B	109.2
N2—C8—C7	122.43 (13)	C14—C13—H13B	109.2
N2—C8—S1	120.95 (10)	H13A—C13—H13B	107.9
C7—C8—S1	116.62 (10)	C4—C5—C6	120.31 (17)
C7—N1—C6	117.62 (12)	C4—C5—H5	119.8
C11—C10—S1	110.83 (10)	C6—C5—H5	119.8
C11—C10—H10A	109.5	C14—C15—C16	112.72 (16)
S1—C10—H10A	109.5	C14—C15—H15A	109.0
C11—C10—H10B	109.5	C16—C15—H15A	109.0
S1—C10—H10B	109.5	C14—C15—H15B	109.0
H10A—C10—H10B	108.1	C16—C15—H15B	109.0
N1—C7—C8	121.19 (13)	H15A—C15—H15B	107.8
N1—C7—C9	119.08 (12)	C7—C9—H9A	109.5
C8—C7—C9	119.73 (13)	C7—C9—H9B	109.5
N2—C1—C2	120.09 (13)	H9A—C9—H9B	109.5
N2—C1—C6	120.89 (13)	C7—C9—H9C	109.5
C2—C1—C6	119.02 (14)	H9A—C9—H9C	109.5
C10—C11—C12	111.23 (13)	H9B—C9—H9C	109.5
C10—C11—H11A	109.4	C3—C2—C1	119.96 (16)
C12—C11—H11A	109.4	C3—C2—H2	120.0
C10—C11—H11B	109.4	C1—C2—H2	120.0
C12—C11—H11B	109.4	C17—C16—C15	114.54 (18)
H11A—C11—H11B	108.0	C17—C16—H16A	108.6
N1—C6—C5	119.52 (14)	C15—C16—H16A	108.6
N1—C6—C1	121.03 (13)	C17—C16—H16B	108.6
C5—C6—C1	119.45 (14)	C15—C16—H16B	108.6
C13—C12—C11	115.33 (13)	H16A—C16—H16B	107.6
C13—C12—H12A	108.4	C2—C3—C4	121.23 (17)
C11—C12—H12A	108.4	C2—C3—H3	119.4
C13—C12—H12B	108.4	C4—C3—H3	119.4
C11—C12—H12B	108.4	C5—C4—C3	120.02 (17)
H12A—C12—H12B	107.5	C5—C4—H4	120.0
C15—C14—C13	115.44 (14)	C3—C4—H4	120.0
C15—C14—H14A	108.4	C16—C17—H17A	109.5
C13—C14—H14A	108.4	C16—C17—H17B	109.5
C15—C14—H14B	108.4	H17A—C17—H17B	109.5
C13—C14—H14B	108.4	C16—C17—H17C	109.5
H14A—C14—H14B	107.5	H17A—C17—H17C	109.5
C12—C13—C14	112.02 (13)	H17B—C17—H17C	109.5
C12—C13—H13A	109.2		
